# Prognostic value of follicular fluid 25-OH vitamin D and glucose levels in the IVF outcome

**DOI:** 10.1186/1477-7827-8-91

**Published:** 2010-07-28

**Authors:** Georgios M Anifandis, Konstantinos Dafopoulos, Christina I Messini, Nektarios Chalvatzas, Nikolaos Liakos, Spyros Pournaras, Ioannis E Messinis

**Affiliations:** 1 Department of Obstetrics and Gynaecology, Medical School, University of Thessaly, Larisa, Greece; 2 Department of Microbiology, Medical School, University of Thessaly, Larisa, Greece

## Abstract

**Objectives:**

The aim of the present study was to measure serum and follicular fluid 25-OH vitamin D and glucose levels in women who underwent IVF-ET treatment and to further investigate whether the circulating 25-OH vitamin D and glucose levels correlate with IVF success.

**Methods:**

This prospective observational study included 101 consecutive women who underwent 101 IVF-ICSI ovarian stimulation cycles and were allocated to one of the three groups according to their follicular fluid 25-OH vitamin D concentrations. Group A (n = 31) with less than 20 ng/ml, group B (n = 49) with vitamin levels between 20.1 and 30 ng/ml and group C (n = 21) with more than 30 ng/ml vitamin concentration.

**Results:**

Follicular fluid vitamin levels significantly correlated with the quality of embryos in total (r = -0.27, p = 0.027), while the quality of embryos of group C were of lower quality as compared to those of groups A and B (p = 0.009). Follicular fluid glucose levels were lower in women of group C as compared to the respective levels of groups A and B (p = 0.003). Clinical pregnancy rate demonstrated in 14.5% in women of group C and 32.3% and 32.7% in groups A and B, respectively (p = 0.047).

**Conclusion:**

The data suggests that excess serum and follicular fluid vitamin levels in combination with decreased follicular fluid glucose levels have a detrimental impact on the IVF outcome.

## Background

Animal and in vitro studies have shown that 25-OH vitamin D may play a significant role in glucose tolerance through its effects on insulin secretion and insulin sensitivity [1]. Few data exist about the role of 25-OH vitamin D in reproduction. Insufficient levels during pregnancy are potentially associated with increased risk of preeclampsia, insulin resistance and gestational diabetes mellitus [2-4], while experimental data indicate that 25-OH vitamin D insufficiency is critical for fetal development, and especially for fetal brain development and immunological functions [5]. Evidence has shown that 25-OH vitamin D levels did not vary during the phases of the menstrual cycle [6] while endometriosis has been associated with higher serum levels of 25-OH vitamin D [7]. The most advantageous serum 25-OH vitamin D levels for its proper action appeared to be 30 ng/ml approximately [8], but the effects of higher concentrations need to be addressed. However, the association of 25-OH vitamin D levels with embryo quality and IVF outcome in IVF-ET treatments has not yet been thoroughly investigated.

Regarding the glucose level in follicular fluid (ff), a substantial utilization of glucose by the oocyte/cumulus cells complex has been reported [9], suggesting a possible influence of ff glucose in the fertilization process. Glucose is a pivotal metabolite for the cumulus oocyte complex (COC) which provides substrates to the oocyte for energy production [10]. It has been reported that the oocyte is exposed to glucose levels at a percentage between 31-82% of ff concentrations when the latter concentrations are at physiological levels [11]. The environment to which the COCs is exposed during maturation affects oocyte development and therefore subsequent embryonic development [12]. Impact of altering levels of glucose metabolism on oocytes and COCs function are likely to be a major cause for diminished oocyte competence and reduced fertility.

Considering the interactions of 25-OH vitamin D with glucose we aimed to measure serum and ff 25-OH vitamin D and glucose levels in women undergoing IVF-ET treatment in order to investigate whether 25-OH vitamin D in combination with glucose concentrations have any impact on the IVF outcome.

## Methods

From January 2009 to August 2009 a total of 101 consecutive women underwent 101 IVF-ICSI cycles. All women studied, after ovarian stimulation, reached the ovum pick up stage, but only 86 managed to reach embryo transfer. All 101 women were allocated to one of the three groups according to their ff 25-OH vitamin D levels. Group A (n = 31), group B (n = 49), group C (n = 21) with less than 20 ng/ml, 20.1-30 ng/ml and more than 30 ng/ml ff vitamin levels, respectively. The biological rationale for this categorization lays in the fact that physiological serum 25-OH vitamin D levels range between 20 and 30 ng/ml [8]. Upper or lower concentrations have been characterized as hypervitaminosis D and hypovitaminosis D, respectively.

### Study design

Our primary end point was to measure serum and ff 25-OH vitamin D levels in combination with glucose concentrations in women undergoing IVF-ET treatment. Secondly, providing availability of embryological data, we investigated if circulating and ff 25-OH vitamin D levels correlate with the IVF outcome.

### Ovarian stimulation

All women received the same ovarian stimulation protocol. Briefly, ovarian stimulation was performed by administration of recombinant FSH (Puregon^®^, Organon, or Gonal-F^® ^Serono) in a short GnRH agonist protocol. Treatment started on day 2 of every woman's menstrual cycle with a FSH dose of 2-6 ampoules (75 IU each ampoule) depending on the age of every woman. This dose was given for 4 days and then it was modified according to the ovarian response. When at least three follicles reached a diameter of 17 mm, 5000 IU of Human Chorionic Gonadotrophin (HCG) was administered and 34-36 hours later, ovum pick up (OPU) was performed under light sedation.

### Fertilization, cleavage, pregnancy rates and assessment of embryo quality

16-18 hours post-IVF/ICSI the fertilization percent, expressed as ratio of oocytes with two pronuclei (2PN) to the total number of injected oocytes, was scored. Fertilization failure was indicated by the absence of PN. Following cleavage of all normal fertilized oocytes, morphological grade of all embryos was assessed 3 days post-OPU. Morphological grade was scored numerically in terms of the blastomeres (4 to 8 blastomeres), grade of fragmentation (in scale 1 to 4 with the fourth scale representing no fragmentation) and irregularity of blastomeres (in scale 1 to 2 with the second scale representing regular blastomeres). For example, one embryo with 8 regular blastomeres and no fragmentation would be assigned a total sum of 14 embryo score, while an embryo with 5 irregular blastomeres and 10-20% fragmentation (scale 2) would be assigned a total sum of 8 embryo score. For every patient, the cumulative embryo score (CES) was calculated by adding embryo score of each embryo and the mean score of embryo quality (MSEQ) was obtained by dividing the CES with the number of embryos produced.

Three days post-OPU and after assessment of embryo quality a maximum of three selected embryos (depending to the age or to the number of available embryos) were transferred to the uterus.

Clinical pregnancy was defined when an intrauterine sac was seen by ultrasound 3-4 weeks post-hCG.

### Vitamin D and glucose measurement procedure

Blood samples were collected for 25-OH vitamin D and glucose assessment during oocyte retrieval. All blood samples were centrifuged at 1,000 g for 15 min, and serum was stored at -20°C until assayed. Additionally, ff was aspirated from each follicle, pooled from all retrieved follicles and centrifuged (800 g for 15 min) after which it should be clear and not contaminated with blood. The ff was also stored at -20°C until assayed. Serum and ff 25-OH vitamin D was measured by the electrochemiluminescence immunoassay (ECLIA) with analytical sensitivity of 0.2 μU/ml, using a Modular Analytics E170 cobas e 601 Analyzer (Roche Diagnostics GmbH, D-68298 Mannheim), while serum and ff glucose levels by using a colourimetric enzymatic procedure, known as the exocinase method (Olympus Life and Material Science Europa GmbH (Irish Branch), Lismeeham, O' Callaghan's Mills, Co. Clare, Ireland).

### Statistical analysis

Pregnancy rates were compared between the three groups with x^2^-test. Numeric data were normally distributed (one sample Kolmogorov-Smirnov test), and statistical analysis was performed by one way analysis of variance followed by Bonferroni post hoc testing. For correlations between the parameters Pearson's correlation was used. P value less than 0.05 was considered significant. All values are expressed as mean ± SD. The statistical software package used was NCSS 2001 (Number Cruncher Statistical Systems, Kaysville, UT).

## Results

**Figure 1:**
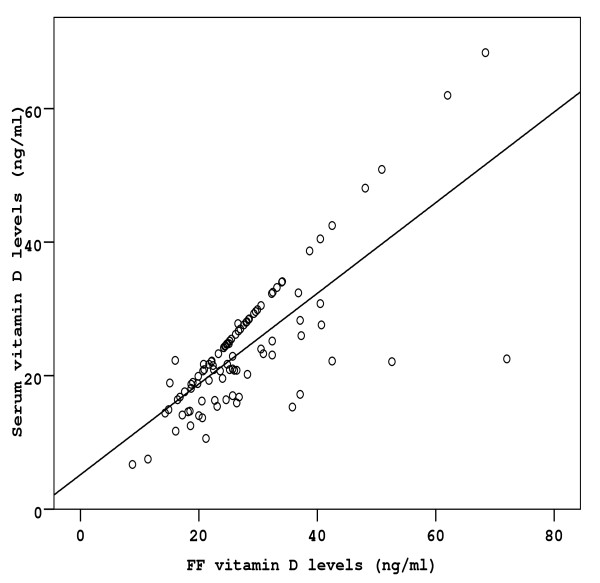
**Correlation between serum and follicular fluid vitamin D concentrations in all women**. The regression fit line is shown.

**Figure 2:**
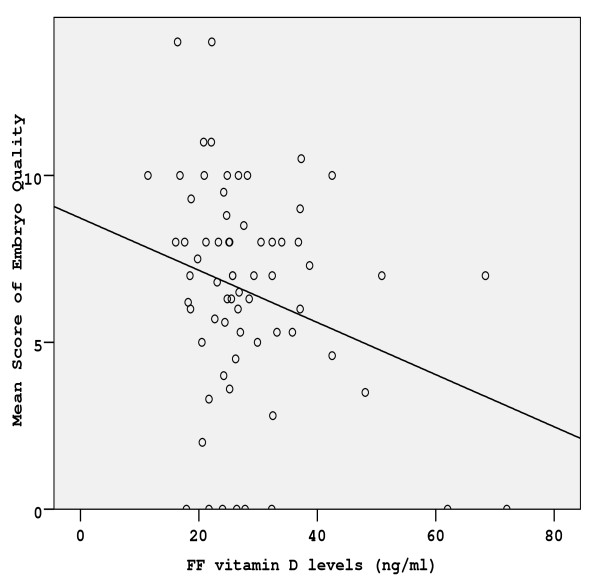
**Correlation between the follicular fluid vitamin D concentrations and the mean score of embryo quality (MSEQ) of the women**. The regression fit line is shown.

Stimulation characteristics in terms of number of ampoules used, number of stimulation days and number of oocytes retrieved, were similar between the three groups (Table 1). In 86 from the total of 101 women studied, embryos were transferred on day 3 post-OPU. The remaining 15 women (group A: 6, group B: 7, group c C: 2) did not have an embryo transfer due to fertilization or cleavage failure. Characteristics, such as age, and BMI, did not differ between the three groups studied (Table 1). Serum vitamin levels of all women studied were significantly correlated with ff vitamin levels (r = 0.79, p < 0.001) (Figure 1), Moreover, ff vitamin levels negatively correlated with embryo quality as depicted in Figure 2 (r = -0.27, p < 0.05). This correlation is reliably reflected in the significant differences in embryo quality between the three groups, with group C demonstrating the lower embryo quality (Table 1). Clinical pregnancy rates of groups A and B were significantly higher as compared to group C (Table 1). The ff vitamin levels of women who achieved pregnancy were significantly lower than those of women that did not become pregnant (22.1 ± 5.5 vs 29.8 ± 8.7 ng/ml, p < 0.05). Furthermore, there were no significant differences between age and BMI values between pregnant and not pregnant women.

**Table 1:** Baseline characteristics (mean ± SD) of the three groups of women studied

	Patient Groups (ff vitamin D, ng/ml)			P value
	A (< 20)	B (20.1 - 30)	C (>30)	
**No of women**	31	49	21	NS
**Age (years)**	35.76 + 5.8	36.35 + 4.5	36.58 + 5.8	NS
**BMI (kg/m2)**	23.8 + 2.6	24.2 + 3.1	24.5 + 3.5	NS
**Serum vitamin D (ng/ml)**	18.7 + 5.74	24.39 + 8.9	30.3 + 11.9	< 0.05
**Serum glucose (mg/dl)**	85.46 + 17.3	86.04 + 18.3	82.3 + 16.2	NS
**ff glucose (mg/dl)**	70.44 + 12.7	66.14 + 12.3	52.82 + 16.7	< 0.05
**No of stimulation days**	10.4 + 2.1	11.6 + 2.6	12.1 + 2.9	NS
**No of FSH ampoules (75 IU each)**	54.5 + 4.8	56.8 + 5.5	57.7 + 5.9	NS
**No of oocytes**	3.9 + 3.2	5.91 + 4.3	6.14 + 5	NS
**CES**	16.29 + 11.2	15.67 + 11.1	15 + 8.1	NS
**MSEQ**	7.96 + 2.6	7.02 + 2.5	5.6 + 3.6	< 0.05
**CPR/woman**	32.3% (10/31)	32.7% (16/49)	14.3% (3/21)	< 0.05
**CPR/ET**	40% (10/25)	38.1% (16/42)	15.8% (3/19)	< 0.05

NS: not significant, BMI: body mass index, ff: follicular fluid, CES: cumulative embryo score, MSEQ: mean score of embryo quality, CPR: clinical pregnancy rate, ET: embryo transfer

A significant negative correlation between ff vitamin levels and ff glucose levels was found (r = -0.25, p < 0.05). Moreover, ff glucose levels were significantly different (p < 0.05) between the three groups with group C demonstrating the lower glucose levels (Table 1).

## Discussion

Our results have shown that the vitamin D levels in the ff were negatively correlated to the quality of embryos and the higher values of vitamin D were associated with lower possibility to achieve pregnancy.

Over the past decade the physiological role of 25-OH vitamin D has been investigated extensively [13], but data regarding its role in human reproduction are scarce. More recently, Ozkan and colleagues [14] reported that replete ff vitamin levels predicts the IVF outcome, proposing therefore the beneficial role of excess vitamin concentrations on endometrial receptivity, since 25-OH vitamin D receptors have been identified in endometrium [15]. The above suggestion seems to contrast with the finding of decreased expression of vitamin D binding protein (VDBP) in the ff of IVF success group reported by Estes and colleagues [16]. This discrepancy is difficult to explain, and more data are needed to elucidate it. However, it is important to note that in the report by Ozkan a significantly higher number of embryos were transferred in the IVF success group compared to the IVF failure group. Although a multivariate regression analysis was performed to show that vitamin D levels in ff independently determined the IVF outcome, the total number of cases was small to draw solid conclusions. Furthermore, in that study [14], the quality of embryos transferred in relation to vitamin D levels was not investigated.

According to the present data, upon increase of ff vitamin levels a small non-significant increase of collected oocytes was observed (Table 1). Although the CES was similar between the three groups, the MSEQ of group C was significantly lower than that of groups A and B (p < 0.05), suggesting that embryos in group C were of poor quality. Taking into account the observed significant negative association between vitamin levels and the quality of embryos (Figure 2) we may hypothize that ff vitamin levels possibly reflect the quality of embryos. It appears that ff vitamin levels, out of the normal range, have a negative impact on the developmental capability of embryos. This effect seems to be even more critical on IVF outcome. Increased ff vitamin levels via the poor quality of produced embryos may result in a poor IVF outcome. The mechanism that vitamin levels exert such an action remains to be investigated, but it appears that glucose levels have a pivotal role in this pathway.

The physiological role of glucose metabolism in regard to reproduction has been determined by several researchers [12,17], but there is no data on the impact of vitamin levels on glucose metabolism. The ff glucose levels were different between the three groups (p < 0.05), suggesting that higher levels of vitamin D corresponded to lower levels of ff glucose. High serum vitamin concentrations probably increase the utilization of insulin, resulting in the proper metabolism of glucose. In contrast to the comparable serum glucose concentrations, significant differences in ff glucose concentrations between the three groups of women were observed. The explanation for this contrast lays in the fact that the lower ff glucose concentrations are influenced not only by the higher vitamin levels (thus increased glucose transportation into granulosa-cumulus cell complex) but also by the presence of other factors which are responsible for follicle/oocyte maturation. It seems that the interaction between extra-ovarian and intra-ovarian factors determine the fate of the follicle and the quality of the oocyte. Nevertheless, glucose concentrations too low or too high are detrimental for oocyte maturation and growth of granulosa and cumulus cells affecting directly the oocyte competence [12,17]. According to our results it seems that high ff vitamin concentrations (>30 ng/ml), probably affecting insulin action resulted in modulation of ff glucose metabolism. This situation seems to be undesirable, since it appears to have directly negative impact on oocyte and embryo quality and subsequently on IVF outcome. Women with vitamin D deficiency (group A) appear to react as women with sufficient vitamin D levels (group B). Women with hypervitaminosis D (group C) during assisted reproduction cycles are associated with poor IVF result, something which in line with the significant higher (p < 0.05) vitamin D levels of IVF failure women as compared to the respective levels of IVF success women. Data about the role of vitamin D in follicle function are lacking in women, however, a study in vitamin D receptor null mutant mice suggested that vitamin D is essential for full gonadal function in both sexes [18].

The measurement of 25-OH vitamin D levels in combination with glucose levels are reported for the first time in women undergoing IVF-ET treatments. It seems that increased ff 25-OH vitamin D levels in combination with the decreased ff glucose levels have a negative impact on embryo quality and therefore on IVF outcome.

## Competing interests

The authors declare that they have no competing interests.

## Authors' contributions

GMA performed the embryological part of the study and drafted the manuscript, KD participated in the clinical part of the study and also participated in its design and coordination of the study, helped to draft the manuscript, CIM helped to draft the manuscript, NC helped to draft the manuscript, NL participated in the assays, SP participated in the assays and IEM participated in the clinical part of the study and also conceived of the study, and participated in its design and coordination of the study and helped to draft the manuscript. All authors read and approved the final manuscript.
